# What does Psychological Well-being Mean for Mexican Late Adolescents? A Study with Natural Semantic Networks in the Post-pandemic Era

**DOI:** 10.11621/pir.2024.0402

**Published:** 2024-12-01

**Authors:** César A. García-Avitia, Sara L. Pérez-Ruvalcaba, Claudia V. Márquez-González

**Affiliations:** a University of Colima, Mexico

**Keywords:** psychological well-being, subjective well-being, psychological meaning, late adolescence, COVID-19 pandemic, natural semantic networks

## Abstract

**Background:**

Well-being is distinguished by its complex and multifaceted characteristics, integrating both objective and subjective components, so each person’s point of view is relevant. In conducting research concerning well-being, it is important account for both age considerations and cultural variability. Additionally, the influence the COVID-19 pandemic should be considered given how it impacted the public including corresponding effects on peoples’ perceptions of well-being.

**Objective:**

The study’s purpose was to analyze the meaning of psychological well-being from the perspective of late adolescents presiding in Colima, Mexico during the post-pandemic era.

**Design:**

A non-experimental, cross-sectional and exploratory research design was used. The *Natural Semantic Networks* technique was used as the instrument of measurement, employing *psychological well-being* as the stimulus concept to study a sample of 112 high school students in late adolescence (92 women, 20 men, ages 17 to 21; average = 17.3). The Natural Semantic Networks research technique enabled the exploration of participants’ subjective meanings associated with well-being.

**Results:**

The analysis identified a set of primary categories of well-being defined by participants including: *health*, both physical and mental; *low-arousal emotions*, such as peace and serenity; *positive affec*t like happiness; *positive relationships*, mainly with family; *security*; *self-control*; and *self-acceptance*.

**Conclusion:**

This research highlights that no single theory fully captures adolescents’ understanding of well-being. Crucial elements considered by the most important theories are missing from participants conceptual frameworks. Additionally, COVID-19 pandemic has affected adolescent perceptions of well-being, especially it its impact on mental health. Peace is also valued as relevant by participants, especially desirable in the midst of emotional turbulence. Results show the need for a more comprehensive perspective on well-being that incorporates specific dimensions of each age group within a cultural and temporal context.

## Introduction

In recent years, the field of psychology has undergone a significant shift, increasingly emphasizing well-being rather than focusing solely on psychopathology (Ryff & Singer, 1996; Das et al., 2020; Kraiss et al., 2022). This transformation reflects a broader understanding that mental health encompasses more than the absence of illness; it also involves positive aspects of human functioning. Seligman and Csikszentmihalyi (2000) have been pivotal in this paradigm shift, advocating for positive psychology, which focuses on cultivating strengths and virtues that enable individuals and communities to thrive. They argue that this approach can enhance well-being and potentially prevent some mental health disorders before they start. Thus, based on these new perspectives, well-being has gained strength as a central construct for psychology and social sciences more generally. For this reason, it has become a topic of global interest, studied in many places around the world (Tay & Diener, 2011).

The interest in evaluating well-being worldwide has led to efforts such as the Gallup World Poll (Gallup, Inc., 2024). This poll, operational since 2005, consistently measures several well-being dimensions using a core survey that includes evaluative judgments of life through Cantril’s Ladder and daily experiences, alongside variables like law and order, health, and standard of living (Lambert et al., 2020). This survey, recently adapted to account for current events and other measurement requirements, is expanded periodically to integrate findings relevant to diverse global perspectives.

Well-being is undoubtedly a relevant issue, but what is it, and what dimensions does it include? For Deci & Ryan (2008), “[well-being] refers to optimal psychological experience and functioning” (p. 1). As Pollard & Lee (2003) have observed the construct of well-being has been approached from different perspectives:

Well-being has been defined by individual characteristics of an inherently positive state (happiness). It has also been defined on a continuum from positive to negative, such as how one might measure self-esteem. Well-being can also be defined in terms of one’s context (standard of living), absence of well-being (depression), or in a collective manner (shared understanding) (p. 64).

Well-being is a multidimensional concept that includes both objective and subjective components, each contributing uniquely to the understanding of human health and happiness. Objective well-being typically refers to measurable aspects of life such as economic stability, health, and safety, which are externally observable and often quantifiable (Diener, 2009). Subjective well-being, on the other hand, concerns individuals’ perceptions and evaluations of their own lives, encompassing emotional reactions and cognitive judgments (Diener, 1984; Diener et al., 1999). Subjective well-being can be further defined to comprise three main components: positive affect (the presence of positive emotions), negative affect (the absence of negative emotions), and life satisfaction (cognitive evaluations of one’s life as a whole). This conceptualization emphasizes the internal and perceived quality of an individual’s experience and personal assessment of their happiness.

According to the Millennium Ecosystem Assessment (2005), an international effort led by the United Nations to evaluate the consequences of changes in global ecosystems for human well-being and to provide a scientific basis for conservation actions aimed at sustainable resources, well-being was defined as a multivariate state comprising five dimensions (Carpenter et al., 2009): health, basic material for a good life, good social relationships, security, and freedom of choice and action. These dimensions of well-being can be considered universal and are useful for approaching this concept, with defined objective and subjective indicators.

The PERMA model, proposed by Martin Seligman (2011), represents a seminal framework in positive psychology, designed to conceptualize and measure well-being. This model identifies five essential elements that contribute to lasting well-being: positive emotions, engagement, relationships, meaning, and accomplishment. *Positive emotions* encompass feelings of happiness and contentment that enhance one’s life experiences. *Engagemen*t refers to involvement in activities that fully absorb and challenge the individual, often leading to experiences of flow. *Relationships* highlight the importance of social connections and support systems that are crucial for emotional and psychological health. *Meaning* pertains to the sense of purpose and significance that individuals ascribe to their lives, often through involvement in something larger than oneself. Finally, *accomplishment* involves the pursuit and achievement of goals, providing a sense of success and fulfillment.

Another important theory defines *psychological well-being*. As outlined by Ryff (1989; 2023a; 2023b), psychological well-being extends beyond subjective contentment and encompasses areas such as personal development and purpose. Psychological well-being comprises six dimensions: self-acceptance, positive relations with others, autonomy, environmental mastery, purpose in life, and personal growth. These dimensions reflect a comprehensive approach to well-being, emphasizing eudaimonic aspects — fulfillment derived from achieving one’s potential and finding meaning in life’s experiences (Ryff & Singer, 1996).

While both subjective and psychological well-being are related to the broader concept of well-being, they differ significantly in scope and emphasis. Subjective well-being focuses primarily on hedonic well-being — immediate happiness and emotional states, whereas psychological well-being addresses more comprehensive aspects of human functioning, incorporating the pursuit of meaningful goals and personal actualization (Ryan & Deci, 2001; Morosanova et al., 2021). Together, these constructs provide a complex picture of what it means to lead a fulfilling life. They highlight the importance of not only ensuring that basic needs are met, and that people feel happy, but also that they grow, thrive, and find value in their daily activities.

A factor that influences how people conceive well-being is their age. The relationship between age and well-being is complex and varies significantly across an individual’s life. Research indicates that subjective well-being often shows a U-shaped curve with age; younger and older adults tend to report higher levels of happiness compared to middle-aged individuals (Stone, Schwartz, Broderick, & Deaton, 2010). Psychological well-being, characterized by feelings of purpose, autonomy, and personal growth, also demonstrates variability with age. For example, older adults often experience higher levels of autonomy and self-acceptance, potentially due to adjustments in life goals and expectations (Ryff, 1989). Moreover, longitudinal studies suggest that while certain aspects of psychological well-being such as personal growth might decline, others like life satisfaction may improve, indicating a complex interplay between different facets of well-being as people age (Charles & Carstensen, 2010).

Regarding adolescence, Avedissian & Alayan (2021) conducted their study to explore the concept of adolescent well-being and to delineate its characteristics, precursors, and empirical indicators through a review of existing literature. Ninety-four articles were examined in the final analysis. Key characteristics of adolescent well-being were identified as autonomy (adolescents’ ability to acquire knowledge and achieve independence), connectedness (maintaining encouraging and supportive interpersonal connections that steer the adolescent toward favorable actions), optimism (assisting the adolescent in remaining optimistic and maintaining a positive outlook despite uncertainties), and competency (aiding the adolescent in positively adjusting to their surroundings and making suitable choices regarding their physical, social, spiritual, and psychological aspects of life). Precursors were categorized into internal and external factors. Internal factors encompassed behavioral, physical, psychological, and spiritual aspects, while external factors covered environmental, economic, educational, leisure, social, and safety and security aspects. For adolescents to achieve well-being, the presence of these domains are necessary, particularly the social aspect.

Late adolescence is a critical developmental stage marked by significant psychological, emotional, and social changes that influence subjective well-being. This period is characterized by increased autonomy from parents, a deeper capacity for abstract thinking, and a heightened focus on peer relationships (Ashfield, 2024; Steinberg, 2014). Adolescents at this stage often experience an intensified quest for identity, as articulated by Erikson’s stages of psychosocial development, which posit that the primary challenge of adolescence is resolving identity versus role confusion (Erikson, 1968).

Subjective well-being during late adolescence is particularly influenced by these developmental challenges. As adolescents strive for greater independence and explore various roles, their well-being may fluctuate due to the instability and pressure of forming a distinct identity (Arnett, 2007; Branje, 2022). Social relationships play a pivotal role; peer acceptance becomes extremely important and can significantly impact an adolescent’s self-esteem and life satisfaction (McMahon et al., 2020; Scholte & Van Aken, 2020).

Furthermore, late adolescents’ cognitive advancements allow them to process emotions more deeply and to consider the future implications of their actions, which can alter their perceptions of happiness and satisfaction. As they develop a more complex understanding of the world and their place within it, their subjective well-being can become more stable or more turbulent, depending on their subjective experiences and coping mechanisms (Erath & Pettit, 2021; Gruhn & Compas, 2020).

In addition to the these various life stage in which a person passes, cultural aspects also influence the perception of well-being (Lambert et al., 2020). Each culture modifies the criteria used to evaluate well-being, and these criteria vary according to context (Díaz et al., 2022). Cultural values profoundly influence the meaning and perception of well-being, reflecting diverse traditions, beliefs, and practices that shape individuals’ conceptions of happiness and fulfillment. According to Diener and Suh (2000), cultural norms dictate the desirable states of living and how happiness is pursued, resulting in diverse expressions of well-being across different societies.

Lambert et al. (2020) emphasize the necessity of including additional dimensions to well-being evaluation to foster a more inclusive perspective on cultural differences. They highlighted the importance of including eudaimonic well-being, which involves utilizing and enhancing one’s best qualities. This concept of well-being, deeply rooted in Aristotle’s virtue ethics, focuses on mastery, life purpose, and personal development. These authors propose incorporating aspects such as connection to nature, mastery, meaning in life, low-arousal emotions, balance and harmony, group relationships, relationships with government, leisure and resilience. These aspects are valued across cultures worldwide as integral components of well-being, extending beyond the perspective of developed Western countries.

Another aspect considered in this research is the effect of the COVID-19 pandemic on people’s perception of well-being and the importance of physical and mental health. This global phenomenon had a sensitizing effect on mental health importance, since public policies and mass media gave it a main place never seen before. The pandemic highlighted mental health as a critical component of well-being due to the spike in instances of anxiety, depression and stress that many people experienced during lockdowns in addition to widespread uncertainty more generally (Fernández-Castillo et al., 2021; Ryff, 2024)

These conditions have been observed since the first months of the pandemic. Pfefferbaum and North (2020) discussed the psychological effects of the COVID-19 pandemic, noting an increase in the prevalence of mental health conditions and emphasizing the need for accessible mental health services. Likewise, Torales et al. (2020) examined pandemic implications for public mental health, suggesting that it gained recognition as an essential pillar of well-being, similar in importance to physical health. Moreno et al. (2020) conducted another relevant study, which analyzed how the COVID-19 pandemic influenced the public’s perception of mental health. Results indicated that the general population began to value it more, recognizing its impact on overall well-being. These studies suggest that the COVID-19 pandemic has transformed public perception and prioritization of mental health, highlighting its integral importance to well-being and underscoring the need to integrate mental health care into public health responses.

Additionally, as this study was conducted among late adolescents in the state of Colima, Mexico, it is important to highlight that Colima has faced a surge in violence perpetrated by organized crime since 2022 (Mexico Daily Post, 2023), significantly impacting public safety. This heightened insecurity has restricted socialization opportunities, altered young people’s perception of risk, and may have influenced their mental health. The unique sociocultural and environmental challenges experienced by adolescents in this region underscore the relevance and pertinence of research in psychological well-being.

After addressing the issues related to well-being, it is important to discuss meaning, a key element in this research. Meaning, as a linguistic representation of an individual’s knowledge and plays a critical role in cognitive processing and behavior. It encompasses the way individuals interpret and mentally organize their experiences, translating complex information into understandable and communicable forms (Lakoff, 1987). This conceptual framework allows people to make sense of their world and act upon it effectively. According to Vygotsky (2012), language and meaning are integral to cognitive development, influencing how individuals learn to perceive, interact with, and respond to their environment. The interplay between linguistic meaning and behavior is evident in how language shapes thought and decision-making processes. For instance, the specificity and structure of language can alter perception and influence behavioral responses, facilitating or hindering communication and social interaction (Boroditsky, 2001). Thus, linguistic representations that constitute meaning not only reflect one’s knowledge but also guides behavior, impacting everything from problem-solving strategies to interpersonal relations.

Meaning is organized into complex structures known as semantic networks. These networks are systems of interconnected concepts where each node represents a word or phrase linked by their semantic relationships, reflecting how meaning is structured in the human mind (Collins & Loftus, 1975). This organization allows for efficient information retrieval and cognitive processing; by activating one concept in the network, a cascade of associations can be triggered, enabling quick access to related ideas (Aitchison, 2003). Semantic networks also facilitate language comprehension and production by organizing knowledge in a way that mirrors natural language usage. For instance, the concept of “dog” might be linked to “animal,” “pet,” and “bark,” illustrating how semantic qualities and relationships are mapped cognitively (Hinton & Shallice, 1991). Thus, semantic networks play a crucial role in linguistic representation of knowledge, underpinning the cognitive mechanisms that enable individuals to understand, communicate, and think critically about their world.

Meaning significantly shapes individual conceptions of well-being and its evaluation. The way people linguistically encode and interpret experiences directly influences their understanding of what constitutes well-being. Vygotsky’s theory of cognitive development suggests that language not only reflects thought but also shapes it (Vygotsky, 1978), implying that linguistic structures used to define well-being impact how individuals perceive and evaluate their own state of wellness. Therefore, studying the meaning of well-being is relevant, and the Natural Semantic Networks technique, described in the methods section below, is particularly useful for this purpose.

## Methods

The research design was non-experimental, transversal and exploratory. The study’s purpose was to analyze the meaning of psychological well-being from the perspective of late adolescents in Colima, Mexico in the post-pandemic era. Data were collected through Natural Semantic Networks (Figueroa et al., 1976; Valdez, 2004). This research technique provided an approach to participants’ subjective meanings of well-being, offering a deeper understanding of perceptions and belief systems from a more natural perspective, less imposed by the researchers.

### Participants

112 respondents took part in this research, 92 women and 20 men, ages 17 to 21 (average = 17.3). All participants were late adolescent students in their last year of high school. The type of sampling was non-probabilistic for convenience. The inclusion criteria were: that the participants were students in their last year of high school in the state of Colima and that they (if they were of legal age) or their parents (if they were minors) gave their informed consent.

### Procedure

The Natural Semantic Networks technique, developed by Figueroa et al. (1976), is a research method used to explore the mental representations individuals have of specific concepts. This technique involves analyzing associative networks that participants create when prompted with a target word, revealing the structure and organization of their knowledge. By mapping these networks, researchers can understand the subjective meanings individuals ascribe to different terms, providing insights into cultural, social, and cognitive dimensions of knowledge. Natural Semantic Networks has been used for research on well-being in studies such as those by Denegri et al. (2015) and Flores-Cano et al. (2020).

The procedure involves asking participants to list *defining words* or concepts they associate with the *stimulus concept*, which in this case was psychological well-being *(bienestar psicológico,* in Spanish). After responding to the five words or concepts, participants were asked to list them according to a its importance, giving the value of *1* to the most important and so on, until reaching the number *5*. The technique was administered on paper, in a format that included written informed consent and was completed by participants whose legal guardians had previously provided informed consent for their participation in March 2024 at the University of Colima facilities. The study was carried out in compliance with the guidelines established by Mexico’s Regulations of the General Health Law on Health Research.

After administration, data were transcribed into a MicrosoftExcel file and prepared for analysis, which consists of the following procedures described by García-Avitia & Tello-Miranda (2022) and García-Avitia (2024).

1. *J Value (J)* retrieval: This process involves counting the total number of distinct defining concepts listed by participants, excluding repetitions. The resulting value reflects the semantic richness and breadth of the network.
2. *Dispersion and Agreement Levels* based on *J Value*: These metrics are determined by identifying the minimum and maximum numbers of possible defining concepts in a Natural Semantic Network. This is calculated by multiplying the total number of participants by the number of defining concepts requested for a stimulus concept (in this case 5). The outcome is the maximum possible number of defining concepts, achievable when no concept is repeated across the network. The minimum possible occurs when all participants list the same five concepts. From the maximum possible, subtract the minimum possible to obtain a range, which is then used to calculate a percentage by multiplying the *J Value* by 100 and dividing the result by the obtained range. This calculation yields a dispersion percentage, which can be classified into one of five levels: very low (0-20%), low (21-40%), medium (41-60%), high (61-80%), and very high (81-100%) (García-Avitia, 2024). Conversely, agreement level is inversely proportional to dispersion level (the greater the dispersion of meaning, the lower the agreement within a sample). It is obtained by subtracting 100 from the dispersion percentage and eliminating the negative sign. When there is agreement on the meaning that a group assigns to a concept, it indicates a consensus in their understanding and interpretation (García-Avitia, 2024). The same 5-level classification from very low to very high is used as for dispersion level.
3. *First-level categorization*: This involves integrating the defining concepts of the Natural Semantic Network by creating categories based on relationships of synonymy (e.g., *tranquility* and *serenity*) or shared linguistic roots (e.g., *health* and *healthy*).
4. *Post-Categorization J Value (PCJ)* retrieval: This is calculated by counting the total number of categories and defining words from the first-level categorization.
5. *Frequency of Defining Word* retrieval: This is calculated by counting the number of times a defining word or category appears in the Natural Semantic Network.
6. *M Value* or *Semantic Weight (M)* retrieval: This involves scoring each category or defining word in the Natural Semantic Network according to the hierarchy of importance assigned by each participant and then summing all results. This requires multiplying the appearance frequency of each category or defining word by the value corresponding to the order assigned (in this case, words assigned number 1 are multiplied by 5, those assigned 2 by 4, and so on, down to those placed at number 5, which are multiplied by 1). This value estimates importance assigned to each category or defining word. It is important to emphasize that the use of natural numbers does not imply that *M value* has an interval measurement level as it remains ordinal data.
7. *SAM Set* retrieval: This is obtained by identifying the 10 or 15 categories and defining words with the highest M Value, representing the core of the Semantic Network. It should be noted that the number of categories and words chosen, whether 10 or 15, is arbitrary, and the usefulness of delimiting this set lies in the ability to concentrate network analysis on what was most relevant at group level. In this technique, *SAM* means *Semantic Association Memory*.
8. *FMG value*: This is obtained by assigning the highest M value of the SAM set a percentage of 100 and then using a rule of three to obtain the rest of the FMG values proportionally. These values are useful to identify proportional semantic distance between each word or category.
9. *Second-level Categorization*: Categories or defining words of the *SAM set* are grouped according to an associative criterion, such as hypernymy/hyponymy related to a semantic field (e.g., *peace*, *calm*, and *tranquility* are grouped into a category called *peace*) (García-Avitia & Tello-Miranda, 2022).
10. *M Value* retrieval by *Second-level Categories*: This is obtained by summing all M values of categories or defining words grouped in the same S*econd-level category*, expressed as ΣM (García-Avitia & Tello-Miranda, 2022).

## Results

To begin the presentation of results on what *psychological well-being* means to participants, the J Value of the network, which represents group’s semantic richness, was J = 235. Considering that there were 112 participants and that 5 defining words were requested, the minimum possible spread was 5 and the maximum possible spread was 560. The operation described in point two of the procedure was performed to obtain the range of defining words, and result was 555 (560–5 = 555). With range value, it was possible to obtain dispersion level (42.3%) and agreement level (57.7%), both remaining at the medium level.

With post-categorization, the J Value was reduced to a score of PCJ = 139. The highest M Value represented was a category defined as *peace*, which also includes defining words such as *calm*, *serenity* and *tranquility*, with an M Value = 199 to which an FMG Value of 100 was assigned. This concepts category is also the one with the highest frequency in the entire semantic network, with n = 67. Second place goes to *mental health* category with an M Value = 187 and a frequency of n = 41, which places it close to first place with only 12 points less in the M Value and a FMG = 93.97. Third place goes to *physical health* category with an M Value = 177, FMG = 88.94 and a frequency of n = 46, which places it close to second category with only 10 points less in the M Value and even a higher frequency.

From the fourth category onwards, the M value scores and frequencies decrease noticeably, so the first 3 categories are clearly the most relevant. Data of the 15 categories or defining words and categories of the SAM Set are shown in *Table 1*.

**Table 1:** Categories or defining words of the SAM Set of psychological well-being meaning

Hierarchy	First-level categories	n	M	FMG
1	Peace	67	199	100
2	Mental health	41	187	93.97
3	Physical Health	46	177	88.94
4	Stability	30	100	50.25
5	Security	22	66	33.17
6	Happiness	23	61	30.65
7	Emotions	16	50	25.13
8	Mind	13	47	23.62
9	Being well	13	41	20.60
10	Family	11	39	19.60
11	Communication	10	29	14.57
12	Emotional intelligence	8	29	14.57
13	Self-esteem	10	26	13.07
14	Being well with oneself	8	25	12.56
15	Self-control	8	24	12.06

*Note. n = frequency. M = M value (semantic weight). FMG = proportional semantic distance.*

**Figure 1.**
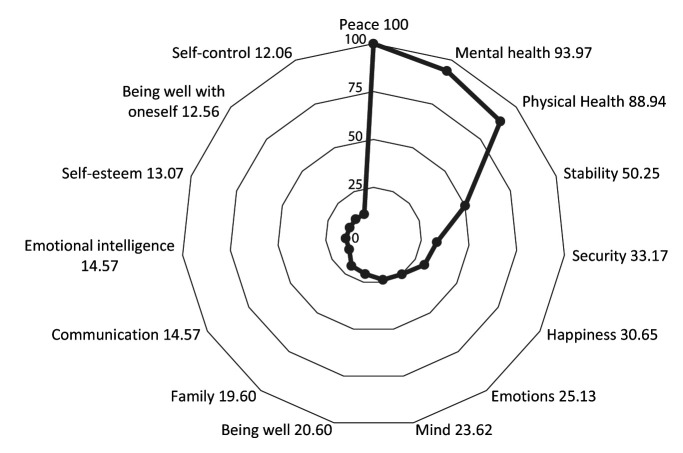
FMG values of the SAM set of psychological well-being

FMG values can be presented using radial diagrams, as shown in *Figure 1*. This facilitates observation of groupings or segmentation within the SAM set, that collectively represent meanings that share similarities or have comparable attributes (Aguilar, 2024). The most significant categories that define psychological well-being are *peace*, *mental health* and *physical health*. A second level is constituted only by the *stability* category. At the third level we can group *security*, *happiness*, *emotions*, *mind* and *being well* categories. A fourth level includes *family*, *communication*, *emotional intelligence*, *self-esteem*, *being good with oneself* and *self-control*.

A second level categorization was carried out with the 15 categories and words included in the SAM Set, which accounted for theoretical proposals on well-being presented in the introduction, resulting in seven groups: *health* (Σ of M = 411), *low-arousal emotions* (Σ of M = 299), *positive affe*ct (Σ of M = 152), *positive relationships* (Σ of M = 68), *security* (Σ of M = 66), *self-control* (Σ of M = 53) and *self-acceptance* (Σ of M = 51). *Table 2* presents these categories organization, as well as their hierarchical position and the M Values per second-level category.

**Table 2:** Second-level categories of the SAM Set of psychological well-being meaning

2nd level hierarchy	2nd level categories	Σ of M	1st level hierarchy	1st level categories	M
1	Health	411	2	Mental health	187
			3	Physical Health	177
			8	Mind	47
2	Low-arousal emotions	299	1	Peace	199
			4	Stability	100
3	Positive affect	152	6	Happiness	61
			7	Emotions	50
			9	Being well	41
4	Positive relationships	68	10	Family	39
			11	Communication	29
5	Security	66	5	Security	66
6	Self-control	53	12	Emotional intelligence	29
			15	Self-control	24
7	Self-acceptance	51	13	Self-esteem	26
			14	Being well with oneself	25

*Note. Σ of M = Sum of M values of each second level category. M = M value (semantic weight).*

## Discussion

Health had the most important meaning among second-level categories, with 3 of the 15 defining categories of the Sam Set included and a joint semantic weight of Σ of M = 411. The fact that health has the greatest relevance, and with a notable difference of 112 points, makes it clear that participants’ conception of well-being is similar to the Millennium Ecosystem Assessment (2005). It is important to emphasize that in this dimension not only physical health would be considered but also mental health. It is quite significant that mental health appears in second place among the 1st level categories, even higher than physical health. This may represent a greater sensitivity among today’s youth toward mental health’s importance to well-being. This may also have been influenced by the emphasis that has been placed on mental health in recent years following the COVID-19 pandemic and public opinion changes (Moreno et al., 2020; Pfefferbaum and North, 2020; Torales et al., 2020).

Another striking aspect is that peace is in first place among second level categories. Lambert et al. (2020) mention that cultures other than those of developed Western countries highly value low arousal emotions, such as tranquility, serenity and peace, and not only joy or those of greater intensity, as the hedonistic perspective of well-being considers (Diener, 1984). Although health surpassed peace and serenity during the second level categorization, the importance given by participants is notable. The emotional instability that pandemic brought, combined with the developmental challenges at this stage (Erath & Pettit, 2021; Gruhn & Compas, 2020), could influence adolescents to consider peace and serenity desirable, which also can be interpreted as the absence of negative emotions (Diener, 1984; Diener et al., 1999).

As expected, the category of happiness and positive emotions also appear among the meanings that adolescents have of psychological well-being (Diener, 1984; Seligman, 2011). However, there is a notable distance between these aspects and the categories at the top, such as health and peace. Likewise, positive relationships are something that also appears among the most important meanings of psychological well-being from the perspective of the participants (Avedissian & Alayan, 2021; Carpenter et al., 2009; Millennium Ecosystem Assessment, 2005; Ryff, 1989; Seligman, 2011). However, it is striking that peer relationships, such as friendships, do not appear among the most relevant categories; instead relationships with family are highlighted. This contrasts with what theories suggest about the relevance of peer relationships at this stage (McMahon et al., 2020; Scholte & Van Aken, 2020). These aspects of socialization could have been affected by the COVID-19 pandemic, since this group experienced contingency and the impossibility of interacting with peers at the beginning of their adolescence. This hypothesis would have to be verified through future studies that can explore the direct influence of the pandemic on the well-being of adolescents.

After positive relationships, the category that obtained fifth place was security. This is a dimension considered relevant in well-being models such as those proposed by Carpenter et al. (2009) and the Millennium Ecosystem Assessment (2005). It is usually considered more as an external factor that influences subjective and psychological well-being (Avedissian & Alayan; 2021). However, the context of Colima, Mexico has been notoriously violent in recent years, with the presence of organized crime that has caused the city of Colima to be considered the most violent in the world due to its homicide rate (Cure Violence Global, 2023). This context may be influencing adolescents to give it greater relevance to security than other dimensions that theories usually consider.

Finally, self-control and self-acceptance appear as the sixth and seventh categories, respectively. Self-control is part of the environmental domain dimension in Ryff’s (1989) theory of psychological well-being, as is self-acceptance. These results are consistent with that author’s perspective. However, in relation to psychological well-being, dimensions such as life purpose, autonomy and personal growth do not appear to be as relevant to the meaning of well-being for the participants.

## Conclusion

One of the contributions of this research is that it demonstrates a single theory is insufficient to encompass adolescents’ understanding of well-being. Adolescents associate well-being with various dimensions including those of objective well-being, subjective well-being, and psychological well-being. Notably, several dimensions of psychological and subjective well-being, such as autonomy, purpose in life, personal growth, peer relationships, competence, and optimism, are absent from their semantic networks. This absence suggests that adolescents may not fully recognize these aspects as integral components of their well-being.

Furthermore, the COVID-19 pandemic may have significantly influenced adolescents’ perspectives on well-being, placing unprecedented emphasis on mental health. Also, it appears that emotional instability experienced during the pandemic has heightened the value adolescents place on peace and serenity, marking these as highly desirable states amidst ongoing uncertainty. Likewise, in the context of violence, security has been considered relevant to well-being. Contributions by this research serve to show the need for a more comprehensive well-being perspective that incorporates specific dimensions of each age group located in a cultural and temporal context.

## Limitations

The first limitation of the study was its limited sample. Furthermore, it was an exploratory study that cannot be generalized. Larger studies are required in different populations to have a more precise perspective. Also, it cannot be certain that COVID-19 pandemic changed well-being perception since there is no prior study.
